# Joint Optimization of Multiprocess Routes and Layout for Low Entropy Flexible Facility

**DOI:** 10.1155/2021/3972772

**Published:** 2021-10-25

**Authors:** Hongtao Tang, Senli Ren, Weiguang Jiang, Jiajiong Liang, Qingfeng Chen

**Affiliations:** ^1^College of Mechanical Engineering, Zhejiang University of Technology, Hangzhou 310023, China; ^2^Xinjiang Institute of Technology, Aksu, Xinjiang 735400, China; ^3^Zhejiang Huizhi Logistics Equipment, Hangzhou 311501, China

## Abstract

Facility layout is not only the premise of production, but also a breakthrough for manufacturing industry to realize energy saving, environmental protection, and low entropy development. On the one hand, considering the interaction between product process routes and facility layout, a joint optimization model is proposed. The model aims to minimize the total logistics cost and consider the global optimization of facility layout and process route planning. On the other hand, considering the application of low entropy concept in facility layout, the analytic network process (ANP) is used to evaluate the low entropy layout. In the choice of the final facility layout, the algorithm results and expert knowledge are considered comprehensively to make up for the shortcomings of the model in the design of qualitative indicators. The algorithm innovation of this paper is to use genetic algorithm (GA) and particle swarm optimization (PSO) to search the solution of product process routes and facility layout simultaneously, to ensure the overall optimal solution of the two decision variables. Finally, an example is given to compare the joint optimization results with the independent optimization results, and the effectiveness of the joint optimization method is verified.

## 1. Introduction

With the continuous upgrading of modern manufacturing industry, enterprises are faced with a series of problems such as high energy consumption and high fluctuation when meeting dynamic demands of customers. As a result, the corresponding workshop logistics costs increase, the production state is unstable, and useless energy consumption leads to the increase of production costs, which seriously affects the sustainable development and core competitiveness of enterprises.

Facility layout design is a key content of production process improvement. It mainly refers to the reasonable arrangement of production factors in a limited space according to the enterprise's own production plan and business philosophy, to satisfy the space required by production factors in the whole process of production and generate the maximum economic benefits [1]. Therefore, facility layout is a breakthrough of manufacturing industry to achieve cost saving, efficient production, and low entropy development. According to the research conducted by Tompkins et al. [2], about 20%–50% of operating costs are usually allocated to facility planning and material handling. The effective facility layout design can reduce 10%–20% of operating costs, but ineffective facility layout will increase the material handling costs by about 30% [3].

In this paper, a low entropy facility layout is proposed, which is low-carbon, energy-saving, and high-anti-interference layout, without increasing the investment of enterprise equipment and resources. In 1865, Clausius proposed the concept of entropy, which represents the degree of chaos in a system [4]. As for the low entropy facility layouts [5], researchers mainly focus on transforming the principle of low entropy into the setting of multiple objectives or multiple evaluation indicators of facility layout. For example, Xiao et al. [6] designed a modified systematic layout planning (SLP) method based on the concept of low carbon, which greatly improved the comprehensive performance of workshop logistics. Zeng and Shen [7] converted material handling costs, workshop nonlogistics relationship, and workshop area utilization rate into multiobjective facility layout model and saved it with heuristic algorithm. Zheng [8] established an accurate facility layout model based on low entropy theory and workshop layout requirements and solved the model by using genetic simulated annealing hybrid algorithm. Chen [9] applied physical entropy to facility layout planning and judged the flexibility and anti-interference of the workshop by calculating the entropy value of the workshop. In this paper, a genetic algorithm based on elite strategy and adaptive crossing-mutation operator is designed to solve the multiobjective low entropy facility layout model.

Scholars from various countries have conducted many studies on various facility layout types [10]. For facility layout in dynamic environment, robust layout planning pursues the optimization of layout objective function in the whole period [11], which is suitable for factories with difficult facility location changes. For example, based on quadratic allocation problem for robust layout planning, Lee et al. [12] proposed a mathematical model for facility layout design in a stochastic dynamic environment. However, the assumption of equal equipment area in the model does not meet the actual production demand. To solve the problem of unequal area facility layout (UA-FLP), Wu and Appleton [13] designed a GA with tree structure to represent a layout and considered the material flows and free spaces between different facilities in the evaluation function. Asl and Wong [14] developed an improved particle swarm optimization (PSO) algorithm, which applied two local search methods and a facility exchange method to improve the quality of the solutions and prevent falling local optimality. Peas et al. [15] introduced two algorithmic approaches to address the UA-FLP: a basic GA and a GA combined with a decomposition strategy via partial solution deconstruction and reconstruction. Liu et al. [16] used a modified multiobjective PSO to solve UA-FLP. Through this innovative heuristic algorithm, the local search based on Pareto optimality and the global optimal search based on the niche technology were combined to obtain the Pareto optimal solution of unequal area facility layout problem. Liu et al. [17] introduced the prominent elastic potential energy based on the overlapping depth of facilities into the layout system to solve the overlapping problem among departments and implemented the gradient method with self-adaptive step size and department deformation strategy to deal with the nonoverlapping constraints in the algorithm solution.

During the transformation from traditional process planning to flexible process planning, flexible process and facility layout influence each other under dynamic environment. Zhou et al., Ma and Cai, and Zha et al. [18–20] proposed a robust layout design method with unequal area for the dynamic facility layout. Li et al. [21] and Kamal and Singh [22] proposed a robust facility layout model considering the impact of product demand changes on process route selection. In the above studies, the multiprocedure route planning was generally taken as the parameter affecting the facility layout in the model, but the interaction between them was ignored. This paper proposed a joint optimization model of multiprocess routing and unequal area facility layout based on robust layout method.

In the study of joint optimization problem [23], there are two methods to solve the problem: one is to carry out integrated coding for nonindependent variables; the other method is to decompose the joint optimization problem into several independent optimization problems and solve them in stages by metaheuristic method. Wen et al. [24] proposed the integrated optimization method of process route planning and facility scheduling. Li et al. [25] proposed an integrated optimization model of process planning and scheduling that took processing cost and equipment load as the optimization objectives. However, the above integrated optimization solutions ignore the interaction between variables, which theoretically reduces the solution space. Based on the joint optimization problem, an intelligent optimization algorithm with multiple decision variables is proposed in this paper to study the joint optimization model of multiple process routes and unequal area facility layout problem for low entropy.

## 2. Mechanism and Evaluation of Low Entropy Facility Layout

### 2.1. Entropy Theory

Information entropy is widely used in human social sciences to measure the uncertainty of information sources. Physical entropy studies particle states and measures the disorder degree of the system mainly by its microscopic characteristics. Based on the understanding of entropy, this paper adopts physical entropy to evaluate the facility layout [26].

The workshop is regarded as an open system composed of particles, and the facilities and materials in the workshop are regarded as particle *X* in the open system. The energy of each particle in the state of *S* is defined as *E*_*s*_ for the different combined productivity of each facility and material in the workshop. Then, the probability *P*_*X*,*s*_ of the workshop in a certain state (*X*, *s*) is expressed as [27]

(1) $$\begin{array}{c} P_{X,s}=\frac{1}{K}\text{exp}\left(-aX-bE_{s}\right). \end{array}$$

Facility entropy *H* is

(2) $$\begin{array}{c} H=K\left(\text{ln}K-a\frac{\partial \;\ln C}{\partial a}-b\;\frac{\partial \;\ln C}{\partial b}\right). \end{array}$$

*K* is the proportional coefficient, *a* is the particle motion rate, *b* is the energy dissipation rate, and *c* is the entropy factor. With reference to (1) and (2), the workshop entropy evaluation index *H* can be deduced as follows:

(3) $$\begin{array}{c} H=-K\sum_{X,s}P_{X,s}\ln P_{X,s}. \end{array}$$

*X* is the number of particles in the system, that is, the number of facilities and materials; *P*_*X*,*s*_ is the probability of the system in a certain state (*X*, *s*); andln  *P*_*X*,*s*_ is the capacity of the workshop in a certain state. The low entropy index of the workshop shows that the more the facilities and materials in the workshop, the higher the entropy value *H*, indicating more chaotic workshop.

High entropy means that the system will increase the degree of chaos, but it also means that the system will be likely to collapse. Applying the above physical entropy theory to the layout system of manufacturing workshop can measure and evaluate the production state of workshop intuitively. In this paper, entropy evaluation index is derived by using the existing entropy theory to evaluate workshop layout.

### 2.2. Low Entropy Application of Facility Layout

The concept of low entropy emphasizes the goal of balanced, low-carbon, and environmental protection in each energy-consuming production process [28].

At the macro level, the increase of workshop entropy is mainly due to the marketization and competition of manufacturing industry. Uncertain customer demand disrupts the production rhythm of the enterprise, which leads to an increase in the number of products, logistics activities, and cost of replacement and procurement of equipment in the production process. According to the physical entropy formula, the larger the quantity of equipment and materials in the workshop, the greater the entropy value *H*. This shows that the more unstable the production state of the workshop, the higher the degree of chaos.

Referring to Liao's research [29] on intelligent facility layout with low entropy, we estimated approximate entropy of four traditional production modes according to the characteristics of workshop layout. Qualitative evaluation of entropy values of four traditional facility layouts is shown in Table 1. Due to the good flexibility, production efficiency, and lower entropy value, the cell group layout shows better performance than other layout modes in the common traditional facility layout.

### 2.3. Evaluation of Low Entropy Facility Layout based on ANP Method

The low entropy facility layout considers the amount of WIP and equipment. In addition, traditional quantitative evaluation indexes such as material handling cost, time loss cost, workshop area utilization rate, and equipment utilization rate should be considered, as well as qualitative evaluation indexes such as flexibility of facility layout and nonlogistics relationship. This paper uses the expert knowledge to design a set of facility layout evaluation system to carry out a more comprehensive evaluation of the facility layout.

In the selection of evaluation methods, the analytic network process (ANP) is a decision-making method which is adapted to the independent hierarchy structure. The typical ANP decision structure can be divided into control layer and network layer [30]:

1. The control layer includes problem objective and decision criteria. The weight of each criterion can be obtained by using ANP method.
2. The network layer is composed of elements dominated by the rules of the control layer. The elements are interdependent and mutually dominated, which is a network structure with interdependent feedback among the elements.

**Figure 1:**
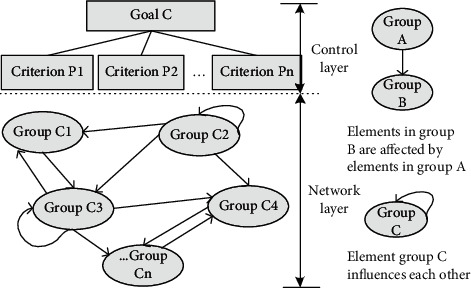
Typical structural model of ANP.

Figure 1 shows the typical ANP structure model. The ANP model makes each decision layer interrelated and can deduce the cross-level interrelation. The top layer of the model is the general goal of the enterprise, and then it is decomposed into various specific criteria and subcriteria layer by layer until the manager can quantify the relative weight of each subcriterion. When we make multiobjective or multicriteria decisions, ANP can be a good solution to these problems. This paper uses the ANP method for the low entropy evaluation system of facility layout, which helps to choose a better layout scheme.

## 3. Joint Optimization Model of Multiprocess Routing and Facility Layout

### 3.1. Problem Description

Workshop background: Take a workshop *A*_0_, for example; there are *P* kinds of products involved, *T* kinds of processes involved, and *N* facilities of different areas involved, named *a*_1_, *a*_2_,…*a*_*N*_.

In this study, two objective functions are defined: (1) the total material handling costs and (2) the mold changing logistics costs. The model needs to satisfy the following constraints:

1. In this paper, the production flexibility is reflected in facilities that can carry out a variety of processes, and processes that can choose a variety of facilities for processing. In the design of process route, the selection of processing facility needs to meet the processing requirements and processing sequence constraints.
2. For UA-FLP issues, ensure that facilities are placed in the workshop and do not overlap with each other.
3. A cell should not have a disjoint set of facilities. This is to ensure that facilities of a particular cell are laid out in contiguous physical locations [31].
4. The number of cells in the workshop is limited, as well as the number of facilities in the cells.

Assume that the material handling distance between facilities is the Manhattan distance between facility centers. Considering the convenience of the description of facility locations, the lower left corner of the workshop is defined as the origin, the *x*-axis coincides with the long side of the workshop, and the *y*-axis coincides with the wide side of the workshop.

**Figure 2:**
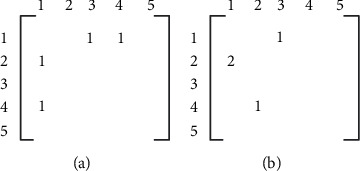
Interpretation diagram of multiprocess route matrix.

Product process route expression: *n* order 0-1 matrix *p*_*n*×*n*,*j*_ represents the process route of product *i*(*i*=1,2,…*m*) in the workshop. Assume that there are five facilities in the workshop; the process route matrix of product *i* is shown in Figure 2. In the process route matrix, the row of each element represents the facility number of the previous process, and the column of each element represents the facility number of the next process.

When product goes in and out of the same facility many times, the matrix shows the process route 2-1-4-1-3 in Figure 2(a). When the same distribution segment appears on the process route several times, the matrix shows the process route 4-2-1-2-1-3 in Figure 2(b).

### 3.2. Nomenclature

#### 3.2.1. Sets

 
*P*: number of product categories in the workshop
 
*N*: number of facilities involved in the process route
 
*O*: number of facilities in the workshop (*O* < *N*)
 
*T*: number of process steps
 
*C*: number of cells

#### 3.2.2. Model Parameters

 
*Z*_*i*_: quantity of orders for product *i*
 
*α*: logistics cost coefficient
 
*B*_*U*_: maximum number of facilities in a cell
 
*B*_*L*_: minimum number of facilities in a cell
 
*l*_*i*_ : length of facility *a*_*i*_
 
*w*_*i*_: width of facility *a*_*i*_
 
*L*: length of workshop *A*_0_
 
*W*: width of workshop *A*_0_

#### 3.2.3. Variables

 
(*x*_*i*_, *y*_*i*_): coordinates of centroid of facility *a*_*i*_
 
*d*_*ij*_: Manhattan distance between facility *a*_*i*_ and *a*_*j*_
 
*d*_*it*_: Manhattan distance between facility *a*_*i*_ and warehouse *a*_*t*_
 
*K*_*i*,*j*_: sequence number of process *j* in the process route of product *i*
 
*O*_*i*, *j*_: facility selected for process *j* in the process route of product *i*
 
*X*_*i*, *c*_: binary variable; if facility *a*_*i*_ belongs to cell *CX*_*i*,*c*_=1; otherwise, *X*_*i*,*c*_=0

### 3.3. Model Formulation

The multiobjective model proposed in this paper aims to minimize the overall cost of material handling and mold changing logistics in the workshop.

Objective function:

(4) $$\begin{array}{c} \text{Z}=\alpha \left(\sum_{i=1}^{p}Z_{i}*P_{n\times n,i}\;\cdot D_{n\times n}+f_{1\times t}\times d_{t\times 1}\right). \end{array}$$

Constraints:

(5) $$\begin{array}{c} k_{i,j}<k_{i,r}\;\left(i=1,2\ldots p;\;j,r=1,2\ldots t\text{ and }j\neq r\right), \end{array}$$

(6) $$\begin{array}{c} O_{i,j}\in \Omega_{j}\;\left(i=1,2\ldots p;\;j=1,2\ldots n\right), \end{array}$$

(7) $$\begin{array}{c} \left(\left|x_{i}-x_{j}\right|>\frac{l_{i}+l_{j}}{2}\right)\vee \left(\left|y_{i}-y_{j}\right|>\frac{w_{i}+w_{j}}{2}\right)\;\left(i,j=1,2\ldots n\right), \end{array}$$

(8) $$\begin{array}{c} \left(l_{i}<x_{i}+\frac{l_{i}}{2}<L\right)\wedge \left(w_{i}<y_{i}+\frac{w_{i}}{2}<W\right)\;\left(i=1,2\ldots n\right), \end{array}$$

(9) $$\begin{array}{c} x_{i}=E,y_{i}=E^{\prime }\left(i\in B;E,E^{\prime }\in R\right), \end{array}$$

(10) $$\begin{array}{c} \sum_{k=1}^{c}X_{ic}=1,\;i=1,2\ldots n,\;k=1,2\ldots c, \end{array}$$

(11) $$\begin{array}{c} \sum_{i=1}^{n}X_{ic}\leq B_{U},\;i=1,2\ldots n,\;\forall c\in C, \end{array}$$

(12) $$\begin{array}{c} \sum_{i=1}^{n}X_{ic}\geq B_{L},\;i=1,2\ldots n,\;\forall c\in C. \end{array}$$

The objective function in (4) is the lowest comprehensive material handling costs and mold changing logistics costs of the workshop. *α* is the logistics cost coefficient based on the unit material handling cost, *Z*_*i*_ is the order quantity of product *i*, and the influence degree of the processing sequence of each product depends on the product demand. The NTH order 0-1 matrix *P*_*n*×*n*,*i*_  is the process route of the product *i*in the workshop; the matrix *D*_*n*×*n*_ represents the Manhattan distance of facilities; and *P*_*n*×*n*,*i*_  · *D*_*n*×*n*_ represents the total material handling costs of the products. Vector *f*_1×*t*_ represents the mold changing frequency of each facility in the workshop; vector *d*_*t*×1_ represents the Manhattan distance between each facility in the workshop and the warehouse; and the *f*_1×*t*_ × *d*_*t*×1_ represents the total mold changing logistics costs of the products in the workshop.

Equation (5) represents the process constraint: process *j* must be carried out before process *r* in the process route of product *i*. Equation (6) represents the facility selection constraint: the product *i* of process *j* must select facility from a collection of its optional facilities *Ω*_*j*_. Equation (7) represents the spatial constraints; that is, there is no overlap between facilities. Equation (8) indicates that the facility shall not exceed the boundary of the workshop. Equation (9) represents the special fixed constraint: facility *a*_*i*_ must be arranged at the coordinate (*E*, *E*′) due to special needs. Equation (10) indicates that when grouping cells, a facility can only belong to one cell. In addition, the size of the cell is limited by (11) and (12), which controls the number of facilities in the cell.

### 3.4. Quasi-Physical Model

**Figure 3:**
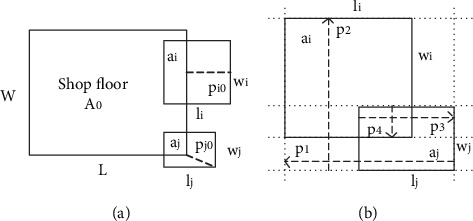
(a) The overlapping depth between facility *a*_*i*_  and workshop *A*_0_; (b) the overlapping depth between facility *a*_*j*_ and workshop *A*_0_.

Inspired by the quasi-physical approach, all facilities and workshop exteriors are assumed to be smooth elastic solids. If there exists overlapping between facility *a*_*i*_ and workshop (Figure 3(a)) or overlapping between facilities *a*_*i*_ and *a*_*j*_ (Figure 3(b)), the computation of the overlapping depth between them is as shown in Figure 3.

If facility *a*_*i*_ and workshop *A*_0_ overlap along the *x*-axis (or *y*-axis) direction or facility *a*_*j*_ intersects the workshop *A*_0_ at a corner (Figure 3(a)), the overlapping depth *p*_*i*0_ and depth *p*_*j*0_ are defined as the minimal displacement distance of facility which moves into the workshop *A*_0_, respectively.

1. If facilities *a*_*i*_ and *a*_*j*_ overlap (Figure 3(b)), the overlapping depth *p*_*ij*_  between them is defined as the minimal displacement distance of facility *a*_*j*_ along the *x*-axis or the *y*-axis, ensuring nonoverlap between facility *a*_*i*_ and *a*_*j*_.

Here, *p*_*ij* _=min{*P*_1_, *P*_2_, *P*_3_, *P*_4_} represents the minimal displacement distance of facility *a*_*j*_, which moves out of facility *a*_*j*_ from the left (*P*_1_), the top (*P*_2_), the right (*P*_3_), and the bottom (*P*_4_) of facility *a*_*i*_, respectively.

(13) $$\begin{array}{c} P_{1}=\frac{1}{2}\left(w_{i}+w_{j}\right)-\left(y_{j}-y_{i}\right), \end{array}$$

(14) $$\begin{array}{c} P_{2}=\frac{1}{2}\left(l_{i}+l_{j}\right)-\left(x_{j}-x_{i}\right), \end{array}$$

(15) $$\begin{array}{c} P_{3}=\frac{1}{2}\left(w_{i}+w_{j}\right)-\left(y_{i}-y_{j}\right), \end{array}$$

(16) $$\begin{array}{c} P_{4}=\frac{1}{2}\left(l_{i}+l_{j}\right)-\left(x_{i}-x_{j}\right), \end{array}$$

(17) $$\begin{array}{c} P_{ij}=\min\left\{P_{1},P_{2},P_{3},P_{4}\right\}. \end{array}$$

When there exists overlapping between facility *a*_*i*_ and facility *a*_*j*_ or between facility *a*_*i*_ and workshop *A*_0_, the extrusive elastic potential energy between them is proportional to the square of their overlapping depth. The extrusive elastic potential energy between them is given by the following equation [32]:

(18) $$\begin{array}{c} E_{ij}=\mu \cdot p_{ij}^{2},\;i,j\epsilon \left\{0,1,2\ldots N\right\},\;i\neq j. \end{array}$$

*μ* is the elastic coefficient. When *j*=0, *E*_*i*0_ is the extrusive elastic potential energy between facility *a*_*i*_ and workshop *A*_0_. Therefore, the total extrusive elastic potential energy is given by the following equation:

(19) $$\begin{array}{c} E\left(X\right)=\sum_{i=1}^{N-1}\sum_{i=i+1}^{N}E_{ij}+\sum_{i=1}^{N}E_{i0}. \end{array}$$

For workshop *A*_0_, if there exists a configuration *X* making *E*(*X*)=0, constraints (7) and (8) are obviously satisfied. The *X* is a feasible solution of the original problem.

## 4. Multiple-Decision-Variable Optimization Algorithm

### 4.1. Algorithm Design

For the algorithm solution of the multi-decision-variable model, when the intelligent optimization algorithm [33] integrates multiple simultaneous search paths into one, the overall search efficiency and diversity will be reduced to a certain extent.

**Figure 4:**
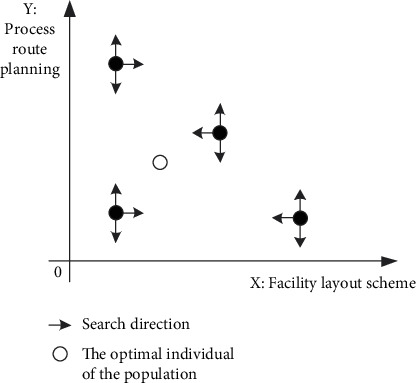
The principle of multi-decision-variable optimization algorithm.

In this paper, an optimization algorithm with multiple decision variables is designed based on population discretization and population rest strategy. In the solution of different dimensions, the optimization algorithm with different characteristics is designed to realize the solution of the multi-decision-variable model. Global search and local search are two characteristics of intelligent optimization algorithm. If global search is used in two-dimensional space, the diversity of solutions is guaranteed, but the efficiency of searching is sacrificed. If local search is used in two-dimensional, the number of solutions may be lost and local optimization may be obtained. When the two dimensions adopt algorithm ideas with different characteristics, the above problems can be solved (as shown in Figure 4). When the individuals of the initial population are scattered enough, the algorithm considers the coordination problem between global search and local optimization.

In the multi-decision-variable optimization algorithm, the selection of each dimension algorithm mainly follows the following criteria:

1. The main characteristics of each dimension algorithm are reflected in the generation (search) rules of solutions
2. The design of intelligent optimization algorithm mainly focuses on the generation of solutions and selection rules

In multi-decision-variable optimization algorithm, any solution of any dimension in each optimization iteration needs to be retained. The lack of iterative solutions will lead to not only the reduction of the overall solution space, but also the failure of optimization iteration.

Among intelligent optimization algorithms, evolutionary operators in evolutionary algorithms include three steps: selection, crossover, and mutation. The crossover and mutation operators can realize good global searching ability of genetic algorithm, and the role of the selection operator is to provide good parent chromosomes for the crossover and mutation steps, that is, to enhance the optimization searching ability [34]. Swarm intelligence algorithm mainly realizes solution search by simulating the behavior of animal groups in nature. The information interconnection among individuals makes the swarm intelligence algorithm be always affected by the known optimal solution in the iteration, so the swarm intelligence algorithm can highlight the feature of local optimization.

Among the swarm intelligence algorithms, particle swarm optimization algorithm and ant colony algorithm are the most widely used, with fast convergence speed and ability to explore the solution space.

In this paper, genetic algorithm (GA) and particle swarm optimization (PSO) are selected to search the solution of process route and facility layout simultaneously, to ensure the best overall optimization effect.

### 4.2. Solution Representation

**Figure 5:**
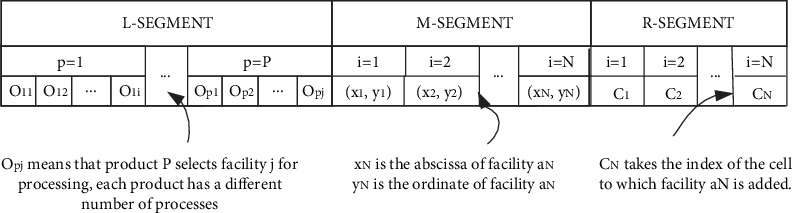
Solution representation for multi-decision-variable optimization algorithm.

Solution representation is the first step in applying intelligent optimization algorithms. The solution representation is designed to solve the proposed model which is depicted in Figure 5.

The left-side segment (L-Segment) encodes the selection of facilities on the process route of product *P*, which needs to meet the process sequence constraints of product *P* (constraint (5)) and process selection on optional facilities (constraint (6)). The middle-segment (M-Segment) encodes the facility layout design of the mathematical model by assigning (*X*_*N*_, *Y*_*N*_) as the facility *a*_*N*_'s location. The location distribution of facilities should satisfy constraints (7)–(9); that is, there is no overlapping interference between facilities, and the fixed location constraints of facilities should be satisfied. The right-side segment (R-Segment) encodes the cell formation through assigning a cell index *c*_*i*_ ∈ {1,2,…*C*} to each facility *a*_*N*_. The grouping of cells for the facility needs to satisfy constraints (10)–(13), which means that one facility can only be assigned to one cell, and the cell must satisfy the limits of size.

### 4.3. Initialization

#### 4.3.1. Initializing the L-Segment

The L-Segment encoding portion can be initialized by randomly transposing the two facility indexes of the same section of the product process route and copying the resulting permutation. The process routing of product *P* needs to satisfy the process sequence constraints and alternative facility constraints.

**Figure 6:**
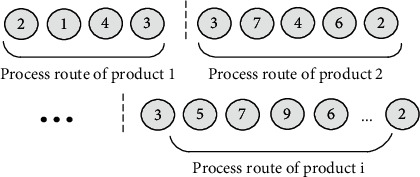
L-Segment initial population coding representation.

The complete process route coding is shown in Figure 6: Each chromosome is divided into *N* segments, and each segment represents the process route of products. The length of each segment is determined by the number of process steps required for the product, and the number represents the facility involved in each process step.

#### 4.3.2. Initializing the M-Segment

1. According to the systematic layout planning (SLP) [35], *M* initial layout schemes were obtained.
2. The location (*X*_*N*_, *Y*_*N*_) of facility *a*_*N*_ is generated randomly, and the initial layout scheme is obtained through the replacement and variation of the facility location. The initial scheme should satisfy the constraints of no overlapped interference between facilities and the placement of facilities not exceeding the workshop boundary and the fixed position of facilities.

**Figure 7:**
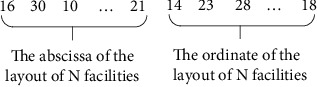
M-Segment initial population coding representation.

Through methods (1) and (2), the initial mixed population coding of facility layout is obtained as shown in Figure 7. The whole code is composed of 2*N* real numbers, and *N* is the number of facilities. The first *n* real numbers represent the *x*-coordinate of each numbered facility in turn, while the last *n* real numbers represent the *y*-coordinate of each numbered facility.

#### 4.3.3. Initializing the R-Segment

1. *C* facilities are randomly designated as the initial facilities of the cell (if the facilities are divided into *C* cells in total, the number of cells is *C*, so *C* facilities are initially selected as the starting positions of *C* cells).
2. When there is an adjacent relationship between the existing facilities in the cell and the assigned facility *a*_*i*_, this means that the cell can accept the addition of facility *a*_*i*_.
3. If there are many cells that can accept facility *a*_*i*_, the cell with the smallest number of facilities is selected for allocation. In the process of cell grouping, the upper and lower limits of the number of facilities contained in the cell should be satisfied.

**Figure 8:**
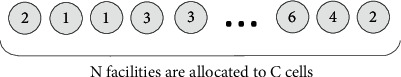
R-Segment initial population coding representation.

This paper develops a simple R-Segment initialization process, as shown in Pseudocode 1. The cell grouping code of the facility is shown in Figure 8: assign *N* facilities to *C* cells, and the index represents the cell number.

### 4.4. Evaluation and Adjustment of Initial Population Dispersion

In Section 4.1, it is mentioned that only when the initial population is sufficiently dispersed, can the binary variable intelligent optimization algorithm not only guarantee the local optimization ability but also not lose the possibility of solution in the solution process. Therefore, it is necessary to define the initial population dispersion mathematically and put forward the optimization method of population dispersion adjustment.

#### 4.4.1. Calculating the Population Dispersion

This paper assumed that the set of points in the two-dimensional solution space is the solution population and the degree of dispersion of points is the degree of dispersion of the solution population. When the dispersion of the initial solution population is larger, the optimal solution after iteration is more likely to be optimal.

Take a two-dimensional solution space, for example; solution *C* consists of *C*_*X*_ and *C*_*y*_, where *C*_*X*_=(*x*_1_, *x*_2_,…*x*_*m*_), *C*_*Y*_=(*y*_1_, *y*_2_,…*y*_*n*_); then, the coordinate of solution *C* in the solution space is (*X*, *Y*):

(20) $$\begin{array}{c} X=Ax_{1}+Bx_{2}+\cdots +\partial x_{m}-\;\left(Al_{1}+Bl_{2}+\cdots +\partial x_{m}\right), \end{array}$$

(21) $$\begin{array}{c} Y=ay_{1}+by_{2}+\cdots +\beta y_{n}-\left(al_{1}+bl_{2}+\cdots +\beta l_{n}\right), \end{array}$$

(22) $$\begin{array}{c} L_{i}\leq x_{i}\leq U_{i},\;\;i=1,2,\ldots ,m, \end{array}$$

(23) $$\begin{array}{c} l_{j}\leq y_{j}\leq u_{j},\;j=1,2,\ldots ,n. \end{array}$$

*A*, *B*,…, ∂;  *a*, *b*,…, *β* are constant coefficient; *U*_*i*_, *L*_*i*_, *u*_*j*_, *l*_*j*_ are the upper and lower bounds of *x*_*i*_, *y*_*j*_. The initial solution population of an optimization is set as *C*, *C*=(*c*_1_, *c*_2_,…, *c*_*m*_). When the distance between any two of the solutions is not less than the real number *ε*, the initial population is considered to be sufficiently dispersed. Let the coordinates of *c*_*i*_ , *c*_*j*_ in the solution space be (*X*_*i*_, *Y*_*i*_), (*X*_*j*_, *Y*_*j*_), *c*_*i*_, *c*_*j*_ ∈ *C*,  *i*, *j* < *m*.

(24) $$\begin{array}{c} \sqrt{{\left(X_{i}-X_{j}\right)}^{2}+{\left(Y_{i}-Y_{j}\right)}^{2}\;}\geq \varepsilon , \end{array}$$

(25) $$\begin{array}{c} \varepsilon =\text{min}\left\{\varphi ,\omega \right\}, \end{array}$$

(26) $$\begin{array}{c} \varphi =\frac{A\left(U_{i}-L_{i}\right)+B\left(U_{2}-L_{2}\right)+\cdots +\partial \left(U_{m}-L_{m}\right)}{2\sqrt{\chi }}, \end{array}$$

(27) $$\begin{array}{c} \omega =\frac{a\left(u_{i}-l_{i}\right)+b\left(u_{2}-l_{2}\right)+\cdots +\beta \left(u_{n}-l_{n}\right)}{2\sqrt{\chi }}, \end{array}$$

*χ*  represents the population size. When the distance between two individuals in the population is too small, the solution of one dimension of the individuals can be adjusted randomly to meet the requirements of dispersion.

#### 4.4.2. Adjusting the Population Dispersion

**Figure 9:**
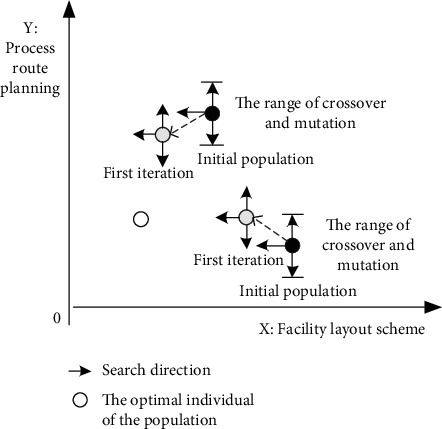
Schematic diagram of adjustment of individuals in a population.

As shown in Figure 9, assume that the black dots in the diagram are two individuals in the initial population. Based on the concept of the initial dispersion of the population and the crossover and mutation range of individuals, it can theoretically cover as many feasible solutions as possible.

In the actual process of each iteration, the solution of one dimension of the individual is constantly approaching the current optimal solution. As shown in Figure 9, the individuals in the population are getting closer to the white point, which makes it almost impossible to find a feasible solution in the iterative process. In order to solve the above problems, population adjustment probability is introduced. If it is judged to be adjusted, then the individual population will conduct mutation or crossover operation in this iteration; that is, feasible solution search will be carried out in the direction of *y*-axis or *x*-axis.

(28) $$\begin{array}{c} b=b_{0}-\left(\frac{b_{0}-b_{1}}{h_{0}}\right)*h. \end{array}$$

*b*_0_ represents the adjustment probability of individuals in the initial population; *b*_1_ is the minimum value of the adjustment probability; *h*_0_ is the maximum number of iterations; *h* is the current number of iterations; and *h*=0 in the initialized population.

When the individual dispersion of the initial population is high, the possibility of losing feasible solutions in iteration is more. With the iteration, the individual population becomes more and more dense; the overlap degree of individual search scope increases; and the adjustment probability decreases accordingly.

### 4.5. Evolution and Renewal

#### 4.5.1. Evolution and Renewal of Process Routes

**Figure 10:**
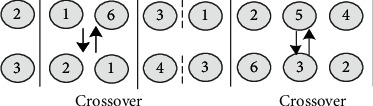
Two-point crossover of multiprocess route population.

*(1) The same chromosome segment is crossover with probability η1*. As shown in Figure 10, the process route is composed of multiple chromosomes of unequal length, which represents the process route of different products. Each chromosome segment can only be crossed between the corresponding chromosomes of the same segment.

**Figure 11:**
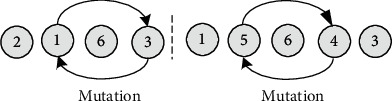
Multisegment mutation of multiprocess route population.

*(2) The same chromosome is mutated with probability η2*. Similarly, since different chromosome segments represent different product process routes, mutation operations are not allowed to occur between different chromosome segments. As shown in Figure 11, the two nodes of the same segment of chromosome with mutation are exchanged to form a new chromosome. In this paper, each segment of chromosome has the same mutation probability; that is, every chromosome has *N* segments and there are *N* times of mutation again, which greatly improves the global search ability.

If the crossover probability *η*1 is too large, it is easy to destroy the existing optimal solution and miss the optimal individual. If *η*1 is too small, it is not conducive to population renewal. Similarly, when the mutation probability *η*2 is too large, the diversity of the population is guaranteed, but the optimal solution is more likely to be broken. When *η*2 is too small, the diversity of the population decreases too fast. Therefore, crossover probability and mutation probability need to be adjusted repeatedly in the actual operation of the algorithm.

#### 4.5.2. Evolution and Renewal of Facility Layout

The update of the solution in PSO is dynamically adjusted based on the optimal position of individual particle history and the optimal position of population history through the change of particle velocity. The equations describing the velocity and position of the particles are given, respectively, as follows [36]:

(29) $$\begin{array}{c} V_{i}=w*V_{i-1}+c_{1}*\text{rand}\left(0,1\right)*\left(p_{i-1}-x_{i-1}\right)+c_{2}*\text{rand}\left(0,1\right)*\left(g_{i-1}-x_{i-1}\right), \end{array}$$

(30) $$\begin{array}{c} x_{i}=x_{i-1}+v_{i}. \end{array}$$

*v* represents the population speed update of each facility layout, and *w* represents the degree of inheriting the population speed of the previous generation. When *w* is large, the particle swarm optimization algorithm has a stronger global search ability. Corresponding to the global search ability of the genetic algorithm mentioned above, the particle swarm optimization algorithm should emphasize its local optimization ability, so *w* should be relatively small. *X* represents a certain facility layout scheme particle, *p* represents the historically optimal particle of each individual, *g* represents the globally optimal particle of the population, and *c* represents the degree to which particles learn from the historically optimal and globally optimal facilities layout scheme. Generally, *c* is set to 2.

The following heuristic layout update strategy is used to update the facility layout population. First, 2*N* free points are randomly generated, which are within workshop *A*_0_ but not within any facility. Choose the facility to relocate based on the following two situations:

(1) *If there are overlapped disturbances in the current facility layout, the facility with the maximum elastic potential energyQ*_*K*_ *is selected as the moving facility*. As particles update their positions, there may be new overlaps between facilities. To make the configuration of the particles feasible, we perform the following heuristic mutation operation steps:

(1) *X*_*i*_=(*x*_*i*1_, *y*_*i*1_, *x*_*i*2_, *y*_*i*2_,…*x*_*iN*_, *y*_*iN*_) is the particle *X*_*i*_ current position.
(2) If the relative elastic potential energy of facilities in the layout is greater than 0, that is, *Q*_*K*_ > 0:
  - The workshop area not occupied by facilities other than facility *a*_*k*_ is defined as the free area.
  - Select the facility with the maximum elastic potential energy among all the facilities to move.
  - In the spare area of the workshop, 2*N* points are randomly formed to place the new central location of facility *a*_*k*_.
  - The elastic potential energy of each facility in the new position is calculated, and the position with the smallest elastic potential energy is selected as the new position of facility *a*_*k*_.
  - In the case of no location change of other facilities, we obtain the corresponding particle update configuration scheme.
  - All the facilities with elastic potential energy greater than 0 are traversed to carry out the above moving processing. When the elastic potential of each facility is less than a minimum value *θ*, the facility location variation process is exited to avoid local optimization.

(2) *If the current layout has no overlap or the degree of overlap is very low, choose the facility with the largest unit material handling cost to move*.

1. By placing the center of the selected facility on randomly generated 2*N* empty spots, we obtain 2*N* new layouts with the other facilities in the same position.
2. If there are no free points in the current layout, we will randomly generate 2*N* points in workshop *A*_0_.

In the legalization of facility layout, the paper used the method of randomly generating blank points to update the layout. Due to the randomness of blank point generation, the iterative process of facility layout legalization algorithm has great uncertainty, which may affect the effectiveness of algorithm implementation.

#### 4.5.3. Evolution and Renewal of Facility Cell Grouping

**Figure 12:**
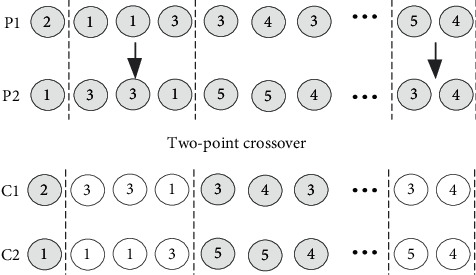
Two-point crossover operation of facility cell group population.

The coding rules of facility cell grouping are not suitable to update through position and speed for particle swarm optimization algorithm, but more suitable to operate for crossover and mutation in genetic algorithm. The evolution and renewal of the grouping population of cells are carried out in the following two ways:

*(1) A two-point crossover operation is applied with a perturbation probability *η*5*. Facilities are randomly selected to exchange cell number, as shown in Figure 12. Assuming that *N* facilities are divided into 5 cells, crossover operation should satisfy the adjacent principle of each facility in the cell and the constraint of cell size.

**Figure 13:**
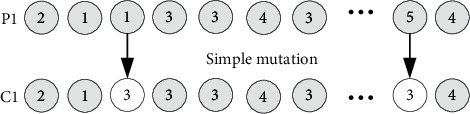
Simple mutation operation of facility cell group population.

*(2) Single point mutation operation with probability *η*6*. As shown in Figure 13, facility has the same mutation probability in each iteration. Too many facilities undergo cell group changes at the same time, rendering the optimal location obtained by updating the plant layout method meaningless. In order to satisfy the constraint that the number of mutations of the target node in each code does not exceed a certain number, the cell mutation probability needs to be continuously debugged. The mutation process of cell grouping should satisfy the constraints of cell size and the number of cells.

Like process route population update, the crossover probability *η*5 and mutation probability *η*6 of facility group population also need to be constantly adjusted in the actual operation of the algorithm, to carry out population update and individual optimization well.

### 4.6. Algorithm Steps and Flow

The overall design process of the multi-decision-variable optimization algorithm in this paper is as follows:

1. Initialize the process route population, facility layout population, and cell grouping population to get the initial solution.
2. Estimate and optimize the population dispersion of the process route population, facility layout population, and facility cell grouping population.
3. The process constraint, layout constraint, and cell size constraint are modified for process route population, facility layout population, and cell group population. That means to satisfy the requirements of specific process sequence, ensuring that there is no overlapping interference between facilities, and the composition of cells should satisfy the constraints of cell size.
4. Calculate the fitness of the initial population and assign the initial value of individual and global optimal population (the initialization assignment of *p* and *g*).
5. Determine whether to perform population adjustment operation. If the conclusion is yes, skip step (7) in the next iteration update step; if the conclusion is no, follow the steps.
6. Carry on the crossover and mutation operation of process route population, and complete the evolution of process route population.
7. Carry out the updating of process route population, facility layout population, and facility cell group population.
8. The process route population, facility layout population, and facility cell group population are modified to satisfy the process constraints, layout constraints, and grouping constraints.
9. Calculate the fitness of the population and update the individual and global optimal population.
10. Determine the maximum number of iterations: if the maximum number is not reached, repeat (5)–(9); otherwise, end the optimization process.

**Figure 14:**
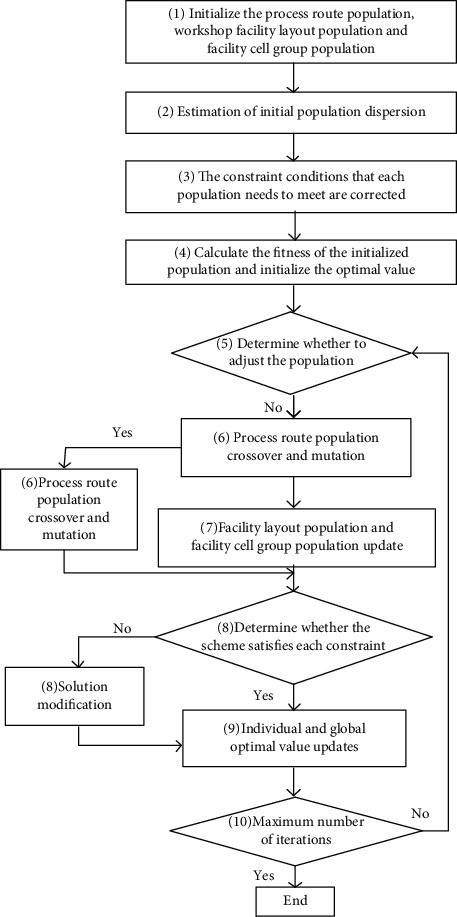
The algorithm steps and flow.

The algorithm steps and flow are shown in Figure 14.

## 5. Case Study

### 5.1. Case Background

The workshop *A*_0_ used for facility layout planning is 90∗50 (m^2^). The historical output and forecast number of products (unit: 10,000 tons) and the process steps of products are shown in Table 2. *A*_0_ takes the AGV as the main mode of transportation, and the power battery of the AGV is lithium battery. Reference [37] shows that the logistics cost coefficient is 0.083 (RMB/m).

There are 15 kinds of facilities involved in workshop production, some of which can carry out different processing processes, as shown in Table 3. The area of each facility is shown in Table 4. The statistics from workshop show that the mold change frequency of all kinds of facility is 0.17 tons per hour, and the mold change frequency of all kinds of facility is 1020 tons per year, calculated based on the working 20 hours per day and 300 days per year (special facility without mold change in the model has 0 tons, such as warehouse).

### 5.2. The Initial Facility Layout Based on SLP

According to SLP [34] (systematic layout planning), the general analysis steps of facility layout are shown below:

1. Analyze the raw materials of the facility
2. Analyze the relationship between logistics and nonlogistics in each facility
3. Determine the comprehensive relationship between facilities
4. Draw the location correlation diagram of the facility
5. Adjust the floor space constraint and nonlogistics relationship of the facility to get some better layout schemes
6. Finally, through the evaluation and adjustment of each scheme, an optimal layout scheme approved by experts was obtained

**Figure 15:**
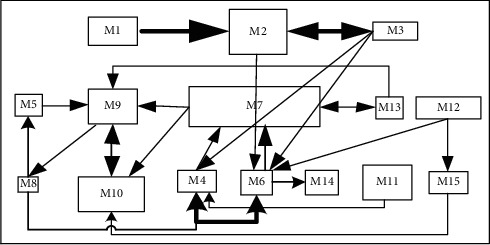
One of the optimal layout schemes obtained by SLP.

The scheme obtained by SLP will be used as the initial solution of the layout scheme algorithm to facilitate the next scheme optimization [38]. The final facility layout scheme is shown in Figure 15. The arrows indicate the logistics relationship between facilities. The thickness of the arrows represents the volume of material flow between facilities, and the direction of the arrows represents the flow direction of materials of facilities.

As can be seen from Figure 15, the disadvantages of the layout scheme obtained by SLP method are as follows: logistics routes between facilities are complicated, and there are problems such as backtracking, which increase system entropy and affect the stability of workshop production system.

### 5.3. Output Analysis

MATLAB is used as the operation environment, with mutation probability of 0.9, crossover probability of 0.1, inheritance coefficient of 0.4 and learning coefficient of 0.6 as the initial parameters of the algorithm. In order to further enhance the global search capability of genetic algorithm, the crossover probability was increased from 0.05 under normal circumstances to 0.1. Similarly, in order to further strengthen the local optimization thought of particle swarm optimization, the inheritance coefficient was lowered from 0.5 to 0.4, while the learning coefficient was increased from 0.5 to 0.6. Regarding the selection of adjustment probability, considering the optimization speed of the algorithm, the initial value 0.7 and the minimum value 0.3 are selected. Finally, with the maximum number of iterations being 300, 30 tests are carried out, and the calculation results are shown in Table 5.

**Figure 16:**
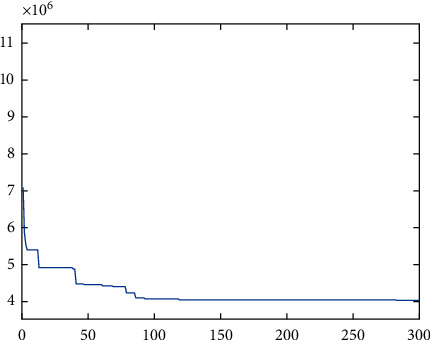
The iterative convergence curve of the optimal results of the joint optimization algorithm.

It can be seen from Table 5 that the minimum annual logistics cost is 4035838 (RMB), and its optimization iteration curve is shown in Figure 16. The optimal process route planning is obtained in Table 6.

### 5.4. Facility Layout Evaluation

Facility layout design is a comprehensive process. It is difficult to fully realize the qualitative and quantitative factors affecting the layout in the algorithm model [33]. The paper proposes a reasonable evaluation framework for facility layout.

Here is a brief introduction to ANP method [39]:

(1) Based on the facility layout background, identify risk factors affecting low entropy layout and construct ANP hierarchical model.
(2) Let the element of control layer in network ANP be *P*_1_, *P*_2_,…, *P*_*m*_; the network layer has element groups *C*_1_, *C*_2_,…*C*_*i*_, *C*_*j*_,…*C*_*N*_; and the element group *C*_*i*_ has elements *C*_*i*1_, *C*_*i*2_,…*C*_*ini*_.
(3) In consideration of the interaction and feedback information of elements in the network structure, *P*_*m*_ is taken as the criterion and *C*_*jk*_  as the secondary criterion, and the influence of element *C*_*ij*_ on element *C*_*jk*_ is compared directly or indirectly by using 1–9 scoring index. The weight vector *W*_*ij*_ is calculated by eigenvalue method, and the hypermatrix *W* is constructed.
  (31) $$\begin{array}{c} W=\left[\begin{array}{ccc} W_{i1}^{\left(j1\right)} & \begin{array}{cc} W_{i1}{}^{\left(j2\right)} & \ldots \end{array} & W_{i1}{}^{\left(jn_{j}\right)}\\ W_{i2}{}^{\left(j1\right)} & \begin{array}{cc} W_{i1}{}^{\left(j2\right)} & \cdots \end{array} & W_{i2}{}^{\left(jn_{j}\right)}\\ \begin{array}{c} \vdots \\ W_{in_{i}}{}^{\left(j1\right)} \end{array} & \begin{array}{cc} \begin{array}{c} \vdots \\ W_{in_{i}}{}^{\left(j1\right)} \end{array} & \begin{array}{c} \ldots \\ \ldots \end{array} \end{array} & \begin{array}{c} \vdots \\ W_{in_{i}}{}^{\left(jn_{j}\right)} \end{array} \end{array}\right]. \end{array}$$
(4) Each element *W*_*ij*_ of hypermatrix *W* is a normalized eigenvector based on a pairwise judgment matrix. By taking element *P*_*s*_  as the criterion, the importance of *C*_*i*_ and *C*_*j*_ of each element group is compared, and the matrix *A* is sorted out. *a*_*ij*_ represents the influence degree of element *C*_*i*_ on element.
  (32) $$\begin{array}{c} A=\left[\begin{array}{ccc} a_{11} & \ldots & a_{1N}\\ \vdots & \ddots & \vdots \\ a_{N1} & \ldots & a_{NN} \end{array}\right]. \end{array}$$
(5) Multiply the matrix *A* by the matrix *W* to get the weighted super matrix *W*′, indicating the importance of each element in the system.
  (33) $$\begin{array}{c} W^{\prime }=a_{ij}W_{ij}. \end{array}$$
(6) To reflect the dependency relationship between elements, the weighted super matrix needs to be stabilized to obtain the limit relative ranking vector.
  (34) $$\begin{array}{c} \underset{N⟶\infty }{\lim}\left(\frac{1}{N}\right)\sum_{K=1}^{N}W^{K}. \end{array}$$
(7) According to the limit hypermatrix, the important factors affecting the low entropy layout of facility were analyzed, and the layout scheme was evaluated.

**Figure 17:**
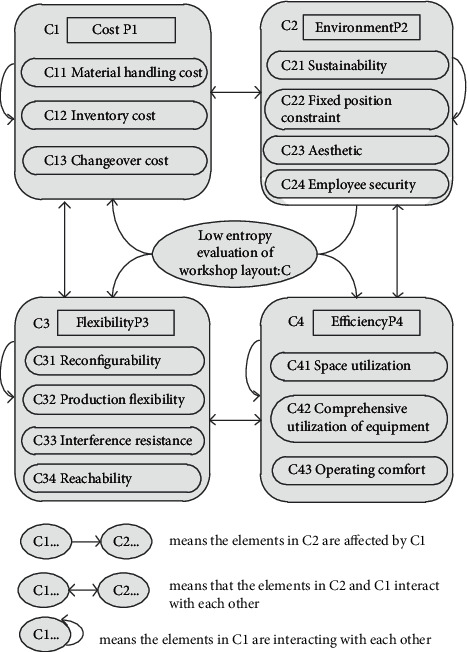
Low entropy evaluation model of facility layout.

In order to comprehensively evaluate the workshop layout optimization, this paper combined the material handling costs and mold changing costs referred to in the model with the proposed low entropy layout evaluation index. The factors affecting the low entropy layout of the facility are analyzed from four aspects: cost, environment, flexibility, and efficiency. Specific indicators are shown in Table 7. According to the interdependence and mutual feedback relationship between various indicators, the low entropy layout evaluation model of the facility was constructed, as shown in Figure 17.

Based on the ANP model of influencing factors of low entropy layout in facility, this paper consulted an evaluation team consisting of 2 project leaders and 4 members. According to the ANP implementation process, calculate the subjective weight of each indicator :

(1) ANP is used to calculate the weight of the four elements in the control layer, judge the consistency of the matrix, and sort out the weight of each criterion, as shown in Table 8:
The eigenvector represents the weight of each element, and the calculation formula is
  (35) $$\begin{array}{c} W_{i}={\left(\prod a_{ij}\right)}^{1/n}. \end{array}$$
Unified disposal is
  (36) $$\begin{array}{c} W_{i}{}^{0}=\frac{W_{i}}{\sum W_{k}}. \end{array}$$
The largest eigenvalue *λ*_max_ is
  (37) $$\begin{array}{c} \lambda_{\max}=\frac{1}{n}\sum_{i=1}^{n}\left(\frac{\sum_{j=1}^{n}a_{ij}w_{j}}{w_{i}}\right). \end{array}$$
The consistency index (CI) is used to measure the consistency of the judgment matrix. The closer the CI value is to 0, the greater the consistency of the matrix.
  (38) $$\begin{array}{c} \text{CI}=\frac{\lambda_{\max}-n}{n-1}. \end{array}$$
The consistency ratio (CR) is calculated to determine whether the matrix satisfies the consistency test:
  (39) $$\begin{array}{c} \mathrm{CR=}\frac{\mathrm{CI}}{\text{RI}}\;<0.1. \end{array}$$
If CR < 0.1, the judgment matrix has satisfactory consistency and no adjustment is required.
According to the above methods, each element of the network layer with mutual influence is judged and compared. For example, element group C1 and element group C2 influence each other, and pairwise judgment should be made for each element group.  Table 9 represents the weight judgment matrix of each factor in element group C1. Tables 10–12 show the importance scores of each element in the C1 group compared to the rest of the elements. Due to limited space, the judgment matrix between or within other element groups is not presented here.
In the C2 element group, C22 has important influence on the C1 element group, while the other factors have minor influence and are temporarily ignored.  Table 13 represents the influence of element C22 on C1 group.
Finally, the hypermatrix *W* is obtained, as shown in Table 14.
(2) Taking control element *P*_*s*_ as the criterion, we compare the importance of *C*_*i*_ and *C*_*j*_ of each element group, obtain the normalized ranking vector *a*_*ij*_ , and sort out the matrix *A*.  Table 15 shows the important relationships between element groups.
(3) Through the MATLAB program processing, the convergence of the limit super matrix is obtained as shown in Table 16.

According to the results of ANP, we can see the following:

1. Cost is worthy of attention. Indicators related to cost shown in the limit supermatrix, such as C11, C112, and C13, account for a relatively high weight, among which the fundamental indicator of C11 accounts for 0.110 and the mold change cost indicator of C13 accounts for 0.097.
2. In terms of environmental criteria, the weight of C21 sustainable development index is 0.091, which is an important evaluation index of this low entropy layout.
3. In the modern production environment, the flexibility and anti-interference of the facility are particularly important. The proportions of the two indexes are 0.093 and 0.090, respectively.
4. In terms of efficiency, space utilization rate and equipment utilization rate affect the flexibility and anti-interference of the workshop, and the weight ratios of indexes are 0.096 and 0.073, respectively.

The ANP method relies on subjective opinions to evaluate the importance of factors that influence facility layout. Due to uncertainty or inaccuracy of the data, it is likely to affect the final conclusions. In such cases, processing techniques based on fuzzy logic may be used. In [40], the authors designed a processing system based on expert experience using fuzzy framework to correctly predict “time to disruption.” In this paper, the ANP method used membership degree to convert expert evaluation into 0–9 numbers.

**Figure 18:**
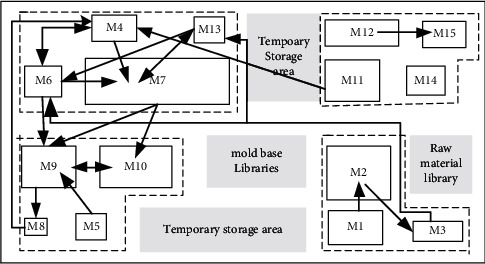
Final facility layout diagram.

In the facility layout, it is desirable to centralize the remaining space and replant it as a system work-in-process staging area and warehouse. Setting appropriate cache area can guarantee the smoothness and stability of production system. The optimized facility layout diagram is shown in Figure 18, which improves the continuity and smoothness of the system production process and reduces the production wait of the equipment in the cell.

## 6. Contrastive Analysis

### 6.1. Facility Layout Optimization of Determined Multiprocess Route

In order to verify the necessity of joint optimization of multiprocess route and facility layout, the process route was taken as the fixed parameter input of the mode. The facility layout optimization of determined multiprocess route is carried out 30 times. As shown in Table 17 the optimal calculation result is 4,527,334 RMB. Compared with the optimal result of joint optimization of multiprocess routes and facility layout, the latter can save logistics cost per year of up to RMB 500,000.

**Figure 19:**
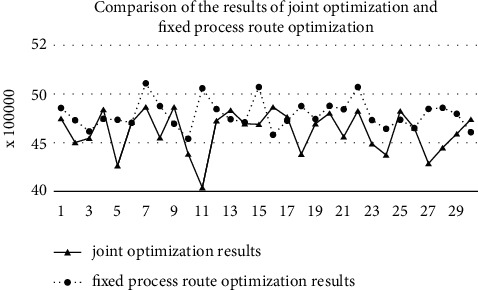
Comparison of the results of joint optimization and fixed process route optimization.

Figure 19 shows the comparison of the results of the two optimization methods. In order to prove that the result of joint optimization is better than that of fixed process route optimization, *T* test of two optimization operation result samples was conducted, and the statistical results are shown in Table 18. The confidence level is 0.95; i.e., *α* = 0.05.

According to Table 18 and (35) and (36), the confidence interval of the comparison between the two optimization results is [−265811, −83747], and the whole confidence interval is less than zero. These show that the result of joint optimization of process and layout is better than that of the fixed process route facility layout.

(40) $$\begin{array}{c} \left({\bar{Y}}_{1}-{\bar{Y}}_{2}\right)\pm t_{df,1-\alpha /2}\sqrt{\frac{S_{1}^{2}}{n_{1}}}+\frac{S_{2}^{2}}{n_{2}}, \end{array}$$

(41) $$\begin{array}{c} df=\frac{{\left(\left(S_{1}^{2}/n_{1}\right)+\left(S_{2}^{2}/n_{2}\right)\right)}^{2}}{\left({\left(S_{1}^{2}/n_{1}\right)}^{2}/n_{1}-1\right)+\left({\left(S_{2}^{2}/n_{2}\right)}^{2}/n_{2}-1\right)}. \end{array}$$

### 6.2. Algorithm Performance Comparison

Taking the above joint optimization results as the optimization results of the multi-decision-variable optimization algorithm, we add the particle swarm optimization algorithm and genetic algorithm to integrate the coding and optimization results of the examples, and the performance of the three algorithms is shown in Table 19. In the actual planning and design, the time is relatively abundant, so the comparison of optimized iteration time is not added in the algorithm performance comparison.

**Figure 20:**
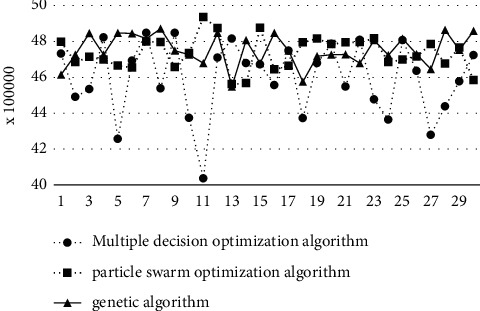
Comparison optimization results of three optimization algorithms.

The average optimization results and comparison of the three optimization algorithms are shown in Figure 20. From the average result, the average optimization result of the multi-decision-variable optimization algorithm is better than that of the particle swarm optimization algorithm and the genetic algorithm. From Figure 20 and Table 20 of the mean and variance results of the three algorithms, the variance of the optimization results of the multi-decision-variable optimization algorithm is large, and the advantages and disadvantages of the optimization results cannot be judged by the single comparison of the average results.

Therefore, it is also necessary to conduct a comparative test of the three samples, and the results are shown in Table 21. From the sample comparison, when solving the facility layout problem represented by the instance object, the solution result of the multi-decision-variable optimization algorithm is better than that of the particle swarm optimization algorithm and the genetic algorithm.

## 7. Conclusion

In this paper, the low entropy oriented flexible facility multiprocess route and layout joint optimization model is applied to the actual facility layout. Through the optimization comparison of joint optimization algorithm, genetic algorithm, and particle swarm optimization algorithm, it is shown that joint optimization algorithm can effectively reduce the facility layout entropy value and improve the layout flexibility, which verifies the feasibility and effectiveness of the method. In the selection of the scheme, this paper combines the algorithm and expert knowledge to evaluate and select the layout scheme. The complex network structure is used to represent the relationship and importance of various factors affecting the low entropy layout of the facility, and the ANP method is used to select a layout scheme.

The joint layout model proposed in this paper assumes that all research cells and facilities are regular rectangles without considering the irregularity of facilities. In future research, the influence of unit equipment irregularity on the modeling solution can be considered to make the joint optimization model more consistent with the actual workshop layout. The low entropy workshop layout evaluation system constructed in this paper adopts the ANP decision method to select the final scheme by combining expert knowledge and algorithm results. However, compared with the actual complex production system, the evaluation structure of this scheme is relatively simple, which needs to be further improved in order to better analyze the influencing factors of low entropy facility layout.

## Figures and Tables

**Algorithm 1:**
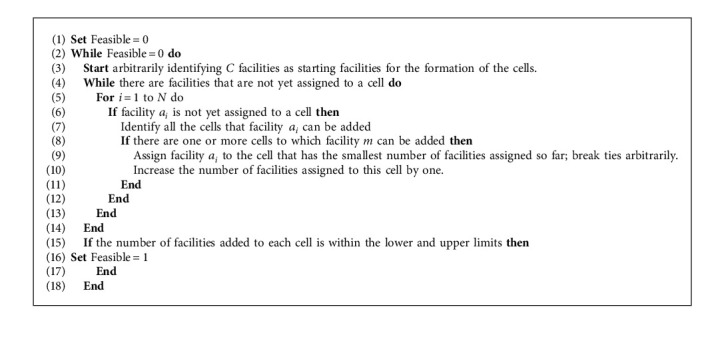
R-Segment initialization.

**Table 1:** Entropy evaluation of each traditional layout.

Layout	Entropy evaluation	*H*=*K∗*∑_*i*=1_^*m*^*p*_*i*_ln *p*_*i*_	Result
Product layout	Suitable for mass assembly line production; poor flexibility; more in stock	The *M* value increases; the system collapse type increases	Middle
Process layout	Suitable for multivariety small batch production; round-trip processing is required	The probability of system collapse in layout increases with the increase of *M* and *p*_*i*_	High
Group layout	Grouping by process similarity, high productivity, good flexibility, and reduced material flow and processing time	Compared with other layout schemes, the quantity of WIP is small and the production process is stable	Low
Fixed production layout	Suitable for large equipment processing, nonprocess layout	The layout does not conform to production requirements	____

**Table 2:** Relevant output and process steps of each product.

Product	Annual prediction	Process
P1	5.55	F1, F2, F3, F6, F7, F8, F9, F10, F11, F12
P2	0.7	F1, F2, F4, F5, F7, F10, F11, F12
P3	3.9	F1, F2, F3, F4, F6, F7, F8, F9, F10, F11, F12
P4	0.75	F1, F2, F4, F5, F7, F10, F11, F12
P5	0.7	F1, F2, F4, F5, F7, F10, F11, F12, F13, F14, F15
P6	0.2	F1, F2, F4, F5, F7, F10, F11, F12
P7	2.4	F1, F2, F3, F4, F6, F7, F10, F11, F15, F17
P8	0.9	F1, F2, F3, F4, F6, F7, F9, F10, F11, F16
P9	0.75	F1, F2, F3, F4, F6, F7, F9, F10, F11, F16
P10	5.1	F1, F2, F4, F6, F7, F13
P11	2.4	F1, F2, F3, F4, F6, F7, F10, F11, F17, F18, F10

**Table 3:** Flexible constraints for each process step.

Process	Optional facility	Process constraint
F1	M1	Before all the processes
F2	M2	Before F6, F7
F3	M3	Before F7
F4	M4, M13	Before F7, F15
F5	M5, M6	Before F7
F6	M5, M6	Before F9, F10, F11, F12
F7	M7	Before F8, F9, F10, F11, F12, F16
F8	M8	Before F9
F9	M4, M13	
F10	M9, M11	
F11	M10	
F12	M9, M11	
F13	M5, M6	Before F7
F14	M6	
F15	M12	After F7
F16	M14	
F17	M4, M13, M15	Before F19
F18	M4, M13, M15	Before F19
F19	M4, M13, M15	

**Table 4:** Length and width of each facility area.

Facility	Length × width	Facility	Length × width
M1	9 × 5.8	M9	9 × 7
M2	10.5 × 9.3	M10	12 × 7
M3	8.2 × 3.5	M11	9 × 7
M4	7.2 × 4.5	M12	12 × 4.5
M5	5 × 4.5	M13	5 × 4.5
M6	6 × 5.2	M14	6.2 × 5
M7	24 × 8	M15	7.2 × 4.5
M8	3.7 × 3		

**Table 5:** Statistics of joint optimization results of multiprocess route and facility layout.

Test	Result (RMB)	Test	Result (RMB)
1	4731474	16	4846871
2	4490077	17	4748016
3	4533120	18	4372415
4	4822324	19	4679011
5	4256539	20	4785634
6	4692628	21	4547632
7	4848016	22	4807756
8	4538258	23	4476465
9	4846871	24	4364277
10	4373556	25	4806732
11	4035838	26	4635284
12	4710130	27	4278424
13	4815480	28	4438258
14	4679011	29	4577632
15	4672415	30	4723246
Average value		4594768	

**Table 6:** Optimal multiprocess route planning.

Product	Optimal process route
P1	M1-M2-M3-M6-M7-M9-M9-M10-M8-M4
P2	M1-M4-M5-M2-M7-M10-M9-M11
P3	M1-M2-M3-M13-M7-M10-M13-M9-M8-M5-M9
P4	M1-M2-M4-M6-M7-M11-M10-M9
P5	M1-M2-M13-M5-M7-M9-M9-M12-M6-M10-M5
P6	M1-M13-M2-M6-M7-M9-M10-M9
P7	M1-M2-M3-M13-M7-M12-M15-M10-M9-M5
P8	M1-M2-M13-M3-M6-M7-M14-M10-M11-M4
P9	M1-M3-M2-M4-M7-M14-M10-M11-M13-M6
P10	M1-M2-M6-M4-M6-M7
P11	M1-M2-M3-M4-M7-M13-M6-M4-M10-M11-M4

**Table 7:** Influencing factors of facility layout evaluation.

Criterion layer	Network layer
Cost	Material handling cost
	Inventory cost
	Changeover cost

Flexibility	Reconfigurability
	Production flexibility
	Interference resistance
	Reachability

Efficiency	Space utilization
	Comprehensive utilization of equipment
	Operating comfort

Environment	Sustainability
	Fixed position constraint
	Aesthetic
	Employee security

**Table 8:** Judgment matrix of control layer criterion.

C	P1	P2	P3	P4	*W*_*i*_^0^
P1	1	6	2	4	0.513
P2	1/6	1	1/3	1/2	0.079
P3	1/2	3	1	3	0.284
P4	1/4	2	1/3	1	0.124

*λ*_max_=4.046, CI = 0.015, CR = 0.017, CR < 0.1.

**Table 9:** The weight judgment matrix of each factor in element group C1.

C1	C11	C12	C13
C11	0.5	0.4	0.25
C12	0.2	0.4	0.35
C13	0.3	0.2	0.4

**Table 10:** Judgment matrix of the influence of element C11 on C2i.

C11	C21	C22	C23	C24	*W*_*i*_^0^
C21	1	1/3	5	4	0.285
C22	3	1	6	5	0.547
C23	1/5	1/6	1	1/3	0.058
C24	1/4	1/5	3	1	0.110

*λ*_max_=4.204, CI = 0.068, CR = 0.076 < 0.1.

**Table 11:** Judgment matrix of the influence of element C12 on C2i.

C12	C21	C22	C23	C24	*W*_*i*_^0^
C21	1	1/3	5	4	0.281
C22	3	1	7	5	0.560
C23	1/5	1/7	1	1/2	0.060
C24	1/4	1/5	2	1	0.098

*λ*_max_=4.107, CI = 0.036, CR = 0.040 < 0.1.

**Table 12:** Judgment matrix of the influence of element C13 on C2i.

C13	C21	C22	C23	C24	*W*_*i*_^0^
C21	1	1/3	5	4	0.281
C22	3	1	7	5	0.560
C23	1/5	1/7	1	1/2	0.060
C24	1/4	1/5	2	1	0.098

*λ*_max_=4.107, CI = 0.036, CR = 0.040 < 0.1.

**Table 13:** Judgment matrix of the influence of element C22 on C1i.

C22	C11	C12	C13	*W*_*i*_^0^
C11	1	7	3	0.669
C12	1/7	1	1/3	0.088
C13	1/3	3	1	0.243

*λ*_max_=3.007, CI = 0.004, CR = 0.006 < 0.1.

**Table 14:** Hypermatrix *W*.

	C11	C12	C13	C21	C22	C23	C24
C11	0.500	0.400	0.250	0.000	0.669	0.000	0.000
C12	0.200	0.400	0.350	0.000	0.088	0.000	0.000
C13	0.300	0.200	0.400	0.000	0.243	0.000	0.000
C21	0.285	0.282	0.282	0.400	0.250	0.250	0.300
C22	0.547	0.560	0.560	0.200	0.350	0.250	0.200
C23	0.058	0.060	0.060	0.100	0.150	0.300	0.350
C24	0.110	0.098	0.098	0.300	0.250	0.200	0.150
C31	0.106	0.059	0.106	0.000	0.254	0.000	0.000
C32	0.523	0.520	0.523	0.000	0.100	0.000	0.000
C33	0.077	0.312	0.077	0.000	0.071	0.000	0.000
C34	0.294	0.109	0.294	0.000	0.575	0.000	0.000
C41	0.200	0.297	0.614	0.163	0.269	0.200	0.297
C42	0.683	0.540	0.269	0.297	0.614	0.600	0.163
C43	0.117	0.163	0.117	0.540	0.117	0.200	0.540

	C31	C32	C33	C34	C41	C42	C43
C11	0.105	0.109	0.109	0.637	0.540	0.637	0.226
C12	0.637	0.582	0.582	0.105	0.163	0.105	0.100
C13	0.258	0.309	0.309	0.258	0.297	0.258	0.674
C21	0.306	0.532	0.532	0.270	0.160	0.138	0.567
C22	0.491	0.270	0.270	0.532	0.467	0.233	0.107
C23	0.125	0.076	0.076	0.122	0.095	0.084	0.061
C24	0.078	0.122	0.122	0.076	0.278	0.545	0.265
C31	0.350	0.200	0.300	0.250	0.060	0.136	0.084
C32	0.350	0.350	0.300	0.250	0.577	0.558	0.545
C33	0.200	0.350	0.300	0.200	0.230	0.078	0.138
C34	0.100	0.100	0.100	0.300	0.133	0.228	0.233
C41	0.200	0.625	0.625	0.136	0.500	0.250	0.300
C42	0.600	0.136	0.136	0.625	0.200	0.500	0.300
C43	0.200	0.239	0.239	0.239	0.300	0.250	0.400

**Table 15:** Matrix of important relationships between element groups.

A	C1	C2	C3	C4
C1	0.400	0.300	0.250	0.300
C2	0.150	0.300	0.150	0.150
C3	0.200	0.200	0.350	0.250
C4	0.250	0.200	0.250	0.300

**Table 16:** Limit hypermatrix.

	C11	C12	C13	C21	C22	C23	C24
C11	0.111	0.111	0.111	0.111	0.111	0.111	0.111
C12	0.072	0.072	0.072	0.072	0.072	0.072	0.072
C13	0.097	0.097	0.097	0.097	0.097	0.097	0.097
C21	0.091	0.091	0.091	0.091	0.091	0.091	0.091
C22	0.053	0.053	0.053	0.053	0.053	0.053	0.053
C23	0.023	0.023	0.023	0.023	0.023	0.023	0.023
C24	0.051	0.051	0.051	0.051	0.051	0.051	0.051
C31	0.055	0.055	0.055	0.055	0.055	0.055	0.055
C32	0.093	0.093	0.093	0.093	0.093	0.093	0.093
C33	0/090	0/090	0/090	0/090	0/090	0/090	0/090
C34	0.048	0.048	0.048	0.048	0.048	0.048	0.048
C41	0.096	0.096	0.096	0.096	0.096	0.096	0.096
C42	0.073	0.073	0.073	0.073	0.073	0.073	0.073
C43	0.048	0.048	0.048	0.048	0.048	0.048	0.048

	C31	C32	C33	C34	C41	C42	C43
C11	0.111	0.111	0.111	0.111	0.111	0.111	0.111
C12	0.072	0.072	0.072	0.072	0.072	0.072	0.072
C13	0.097	0.097	0.097	0.097	0.097	0.097	0.097
C21	0.091	0.091	0.091	0.091	0.091	0.091	0.091
C22	0.053	0.053	0.053	0.053	0.053	0.053	0.053
C23	0.023	0.023	0.023	0.023	0.023	0.023	0.023
C24	0.051	0.051	0.051	0.051	0.051	0.051	0.051
C31	0.055	0.055	0.055	0.055	0.055	0.055	0.055
C32	0.093	0.093	0.093	0.093	0.093	0.093	0.093
C33	0/090	0/090	0/090	0/090	0/090	0/090	0/090
C34	0.048	0.048	0.048	0.048	0.048	0.048	0.048
C41	0.096	0.096	0.096	0.096	0.096	0.096	0.096
C42	0.073	0.073	0.073	0.073	0.073	0.073	0.073
C43	0.048	0.048	0.048	0.048	0.048	0.048	0.048

**Table 17:** Statistical table of optimization results of facility layout considering multiprocess routes.

Test	Result (RMB)	Test	Result (RMB)
1	4837267	16	4567342
2	4714108	17	4708463
3	4601877	18	4856334
4	4728206	19	4725877
5	4718382	20	4858614
6	4688681	21	4824024
7	5085313	22	5047334
8	4856569	23	4715313
9	4678614	24	4627334
10	4527334	25	4718313
11	5035479	26	4635479
12	4826043	27	4828206
13	4724024	28	4838614
14	4693443	29	4778614
15	5047800	30	4593443
Average value		4769548	

**Table 18:** Statistical data of joint optimization and fixed process route optimization.

	Scheme 1: multiprocess route and facility joint optimization	Scheme 2: determining multiprocess route facility layout optimization
Sample mean	$\bar{Y_{1}}=4594768$	$\bar{Y_{2}}=4769548$
Sample variance	*S*_1_^2^=4128058421	*S*_2_^2^=2087072619

**Table 19:** Optimization results of three optimization algorithms.

Multi-decision-variable optimization algorithm		Particle swarm optimization algorithm		Genetic algorithm	
Test	Result	Test	Result	Test	Result
1	4731474	1	4797267	1	**4614781**
2	**4490077**	2	4684242	2	4725643
3	**4533120**	3	4714177	3	4846342
4	4822324	4	**4698206**	4	4725877
5	**4256539**	5	4665382	5	4846871
6	4692628	6	**4653681**	6	4844156
7	4848016	7	4799313	7	**4815480**
8	**4538258**	8	4796569	8	4871334
9	**4646871**	9	4657125	9	4748016
10	**4373556**	10	4734261	10	4725877
11	**4035838**	11	4935479	11	4679011
12	**4710130**	12	4873442	12	4848016
13	4815480	13	4561353	13	**4547632**
14	4679011	14	**4567531**	14	4806732
15	**4672415**	15	4875800	15	4679264
16	**4556539**	16	4644571	16	4846871
17	4748016	17	**4664138**	17	4748277
18	**4372415**	18	4794134	18	4575832
19	**4679011**	19	4815732	19	4718313
20	4785634	20	4784132	20	**4725877**
21	**4547632**	21	4794254	21	4727334
22	4807756	22	4796334	22	**4679011**
23	**4476465**	23	4816642	23	4806732
24	**4364277**	24	4684234	24	4724024
25	4806732	25	**4697731**	25	4815480
26	**4635284**	26	4714451	26	4729677
27	**4278424**	27	4785206	27	4645811
28	**4438258**	28	4675614	28	4863880
29	**4577632**	29	4765241	29	4753263
30	4723246	30	**4583673**	30	4858614
Sample mean **4594768**		Sample mean 4734331		Sample mean 4745701	

**Table 20:** Statistical table of average optimization results of three optimization algorithms.

	Sample mean	Sample variance
Multiple-decision-variable optimization algorithm	$\bar{Y_{1}}$ **=** **4594768**	*S*_1_^2^ **=** **4128058421**
Particle swarm optimization algorithm	$\bar{Y_{2}}$ = 4734331	*S*_2_^2^ = 844401519
Genetic algorithm	$\bar{Y_{3}}$ = 4745701	*S*_3_^2^ = 786938116

**Table 21:** Sample comparison of optimization results between the multi-decision variable optimization algorithm and the other two algorithms.

Confidence coefficient 0.95	Multiple decision variables, particle swarm optimization	Particle swarm optimization, genetic algorithm
Confidence interval	[−220986, −58137]	[−221364, −69129]

## Data Availability

The data used to support the findings of this study are included within the article.
